# The association between pregnancy outcomes and frozen-thawed embryo transfer cycles based on D3 cell count in high-quality blastocysts

**DOI:** 10.3389/fendo.2024.1464313

**Published:** 2024-10-18

**Authors:** Xiang Li, Youman Zeng, Lingling Zhu, Zengyu Yang, Yudi Luo, Jun-Long Jia

**Affiliations:** ^1^ Reproductive Medicine Center, Maternal and Child Health Care Hospital of Yulin, Yulin, China; ^2^ Reproductive Center, No.940 Hospital of Joint Logistic Support Force of the Chinese People’s Liberation Army, Lanzhou, China

**Keywords:** frozen-thawed embryo transfer, high-quality blastocyst, D3 cell count day, pregnancy outcome, single blastocyst transplantation

## Abstract

**Objective:**

To investigate the number of cells in D3-stage embryos of high-quality blastocysts as a contributing factor, to evaluate the clinical pregnancy outcomes in frozen-thawed embryo transfer cycles, and to determine the impact of D3-stage cell count on pregnancy outcomes.

**Methods:**

Patients under 38 years old who underwent frozen-thawed single high-quality blastocyst transfer at our center were selected. Based on the cell count of D3 cleavage-stage embryos forming blastocysts, patients were divided into three groups: ≤6 cells, 7-9 cells, and ≥10 cells. A multivariate regression analysis was used to establish the prediction model, analyzing the impact of different D3 cleavage-stage cell counts on clinical pregnancy outcomes to guide clinical laboratories in selecting blastocysts with the best pregnancy outcomes for transfer.

**Results:**

This study identified a significant association between D3 cell count, blastocyst development stage, and embryo age. Embryos with a higher D3 cell count (≥10) were more likely to reach advanced blastocyst stages and form blastocysts by D5, whereas embryos with fewer D3 cells (≤6) were more likely to form blastocysts on D6. While D3 cell count significantly influenced blastocyst stage and timing of embryo development, no significant differences were observed between groups regarding clinical pregnancy, implantation, or live birth rates. Notably, embryos with fewer D3 cells exhibited a significantly lower miscarriage rate than other groups. Multivariate regression analysis showed a significant correlation between blastocyst stage, embryo age, and D3 cell count, particularly in D5 embryos and more advanced blastocysts. The increased miscarriage rate may be related to lower D3 cell count, and inadequate endometrial preparation was associated with poorer pregnancy outcomes. The type of infertility was also linked to D3 cell count, with secondary infertility patients showing more significant influencing factors.

**Conclusion:**

D3 cell count and related factors play a critical role in pregnancy outcomes during frozen-thawed high-quality blastocyst transfer cycles. Optimizing embryo age, selecting blastocysts at different stages, and refining endometrial preparation protocols are likely to enhance clinical pregnancy and live birth rates.

## Introduction

In assisted reproductive technology (ART), the transfer of a single blastocyst is widely accepted as it is crucial for achieving a successful pregnancy and is also important for the health of both the mother and the baby (1). Recently, novel strategies for embryo selection have emerged, such as delayed imaging (2), preimplantation genetic testing for aneuploidy (PGT-A) (3), and the analysis of gene expression in cumulus cells and metabolite levels in culture media (4, 5). Despite these advances, morphological scoring remains the predominant assessment method for evaluating embryo quality.

Blastocyst formation is a complex and continuous process involving key events such as blastomere proliferation during cleavage, compaction, and the development of the blastocyst cavity (6, 7). Embryonic genome activation, occurring during the 4-cell to 8-cell stage of cleavage, marks a crucial transition in blastocyst development (8). It is widely recognized that the ideal embryo for transfer is an 8-cell stage embryo with uniformly sized blastomeres on Day 3, as deviations in blastomere number can lower implantation rates (9). Blastomere count is thus a critical metric for evaluating blastocyst development, directly influencing its quality and potential for implantation.

The impact of blastomere number on the pregnancy outcomes of blastocyst transfer is debated. Some researchers argue that blastocysts derived from Day 3 embryos with a higher number of cells exhibit greater developmental potential, leading to increased rates of implantation and live births (10, 11). Conversely, other studies suggest that blastocysts derived from fewer blastomeres exhibit comparable implantation and live birth rates, though embryos with faster cleavage rates may have an increased risk of chromosomal abnormalities (12, 13). Therefore, definitive conclusions from research are still lacking.

Research on how the number of cells in D3 embryos affects pregnancy outcomes in frozen-thawed high-quality blastocyst transfer cycles is currently limited. This study aims to reveal the potential impact of high-quality blastocysts from different blastomere counts during frozen-thawed transfer cycles through in-depth analysis. The goal is to establish a feasible predictive model, providing clinicians with more accurate criteria for embryo selection.

## Materials and methods

### Data collection

A retrospective analysis was conducted on infertile couples undergoing frozen-thawed embryo transfer cycles at the Maternal and Child Health Care Center of Yulin from January 2018 to November 2023. The study was approved by the Ethics Committee of the Maternal and Child Health Hospital of Yulin. Before participation, all subjects were informed about the study details and provided written consent.

Data collection included variables such as age, body mass index (BMI), duration of infertility, type and causes of infertility, fertilization method, endometrial preparation protocol, endometrial thickness, implantation rate, clinical pregnancy rate, miscarriage rate, multiple pregnancy rate, and live birth rate.

### Inclusion and exclusion criteria

#### Inclusion criteria

Female age <38 years. Transfer of high-quality single blastocysts post-thawing, defined as blastocysts with a Gardner score of ≥3BB (14). This includes blastocysts with a well-developed blastocyst cavity, a clear inner cell mass, and a well-defined trophoblast layer. Normal endometrial thickness and morphology at the time of transfer. “Normal” endometrial thickness is defined as ≥7 mm, and the endometrial morphology should be trilaminar as observed via ultrasound, indicating a receptive uterine environment for embryo implantation. Normal blastocyst expansion, defined as the blastocyst cavity volume having returned to at least half of its pre-freeze volume, indicating that the blastocyst is in an appropriate state for implantation. Transfer of high-quality single blastocysts that are at the 2PN (two pronucleus) stage, assessed approximately 16-18 hours post-insemination on Day 1.

#### Exclusion criteria

Patients diagnosed with uterine abnormalities including hydrosalpinx, endometriosis, or uterine fibroids. Patients where either partner has a documented chromosomal abnormality. Patients with blastocysts that did not fully expand or collapsed after thawing. Patients undergoing embryo transfer during the cleavage stage. Patients undergoing fresh transfer cycles. High-quality blastocysts developed from Day 3 embryos graded as third level.

### Patient group

A total of 574 FET cycles meeting the above criteria were performed, corresponding to 574 individual treatment cycles. Some patients underwent more than one cycle, but to control for potential patient bias, only the first treatment cycle per patient was included in the primary analysis. Patients were divided into three groups based on the number of cells in the D3 embryos that formed the transferred blastocysts: Group A (≤6 cells, n = 66 cycles), Group B (7-9 cells, n = 381 cycles), and Group C (≥10 cells, n = 127 cycles). The order of transfer was prioritized based on the blastocyst grading for each patient.

### Methods for grading cleavage-stage embryos and blastocysts

According to the “Istanbul Consensus” (9), the grading system for cleavage-stage embryos considers cell number, fragmentation amount, cell size, texture, color, and the uniformity and arrangement of pronuclei. Cleavage-stage embryos are classified into high-quality, moderate-quality, and poor-quality embryos. High-quality embryos have 8-9 cells, less than 10% fragmentation, cell sizes appropriate for the developmental stage, and no multinucleation. Moderate-quality embryos have more than 6 cells, 10%-25% fragmentation, most cells of appropriate size for the developmental stage, and no multinucleation. Poor-quality embryos are characterized by fewer than 6 cells, more than 25% fragmentation, irregular cell sizes, and multinucleation.

The survival rate of frozen-thawed blastocysts is evaluated based on the expansion of the blastocyst cavity within 2-4 hours post-recovery. The quality of thawed blastocysts is assessed using the Gardner grading system (14). Blastocysts with inner cell mass or trophectoderm cells graded as B are deemed usable. High-quality blastocysts are defined as those with inner cell mass and trophectoderm cells both graded ≥B and reaching stage 3 or higher. The high-quality blastocyst rate is the proportion of high-quality blastocysts to the total number of blastocysts at stage 2 or above. The integrity rate represents the proportion of fully recovered embryos to the total number of thawed embryos.

### Embryo vitrification and thawing protocols

In our embryo cryopreservation protocol, we use vitrification to preserve blastocysts on days 5-6. This process involves Cryotop (Kitazato, Japan) and commercial vitrification reagents (VT601, Kitazato). The warming process utilizes the warming kit (VT602) from Kitazato (Tokyo, Japan). We adhere to the manufacturer’s vitrification and warming protocols, as described in previous studies Vitrified blastocysts are thawed and cultured *in vitro* for 2-4 hours before transfer. If blastocysts collapse during warming, the FET cycle is typically canceled or a re-thawing is performed. Outcomes of each individual blastocyst transfer are recorded.

### Preparation of endometrium and embryo transfer

Our reproductive center employs three protocols for uterine preparation before embryo transfer: hormone replacement cycle, natural cycle, and ovulation induction cycle. In the natural cycle, monitoring of endometrial and follicular development starts from day 10 of the menstrual cycle using ultrasound. Luteal phase support therapy begins when the leading follicle reaches 18-24 mm in diameter and the endometrial thickness is ≥8 mm (defined as day 0), with embryo transfers scheduled on days 0, 3, or 5 accordingly. For the hormone replacement cycle, oral estradiol valerate (Tonicare, Bayer, Germany) is initiated on days 2-3 of menstruation until the endometrium reaches ≥8 mm, followed by progesterone injection (20 mg/IU, Zhejiang Xianju) for luteal support, with transfers scheduled on days 0, 3, or 5. In the ovulation induction cycle, letrozole is orally administered for 5 days starting from days 3-5 after menstruation, coupled with human menopausal gonadotropin (HMG) for ovulation induction. Progesterone is administered once the dominant follicle reaches ≥17 mm and the endometrial thickness is ≥8 mm, preparing the endometrium for embryo transfer.

In all cycles, we consistently use COOK catheters manufactured by COOK Medical, headquartered in Indiana, USA, for transferring selected embryos into the uterine cavity under ultrasound guidance. This standardized catheter use ensures methodological consistency and minimizes potential variations that could occur with different catheters.

### Clinical outcomes

In assessing pregnancy outcomes, we analyze several indicators: clinical pregnancy, clinical pregnancy rate, implantation rate, multiple pregnancy rate, miscarriage rate, and live birth rate. The clinical pregnancy rate is calculated as the number of cycles with the presence of a gestational sac and fetal heartbeat at 4 weeks post-embryo transfer, divided by the total number of embryo transfer cycles. *β*-hCG levels are tested on the 11th day after D5/D6 frozen-thawed blastocyst transfer, with levels above 50 mIU/mL considered hCG-positive. Clinical pregnancy is confirmed if both a gestational sac and fetal heartbeat are detected by the 4th week after embryo transfer. In cases where multiple live births occur following single embryo transfer (SET), this is likely due to the natural splitting of a single blastocyst into monozygotic twins, a phenomenon that, although rare, occurs occasionally in assisted reproductive technology (ART). To avoid errors, our embryo transfer process involves strict double-checking by two individuals. Implantation rate is calculated by determining the number of gestational sacs per transferred embryo at the 6th week of pregnancy. Miscarriage rate is computed as the number of miscarriages divided by the number of clinical pregnancies. The rate of multiple births is derived from dividing the number of multiple pregnancies by the number of clinical pregnancies. Live birth rate signifies the proportion of live births per clinical pregnancy.

### Statistical analysis

This study determined the required sample size using G*Power 3.1.9.7 and applied a Chi-square test to assess differences in pregnancy rates across three groups. The statistical parameters were: an effect size (w) of 0.3 (medium), a significance level (α) of 0.05, statistical power (1-β) of 0.80, and degrees of freedom (df) of 2. Based on these inputs, G*Power calculated a total sample size of 108, with approximately 36 frozen-thawed blastocyst transfer cycles per group. This sample size provides 80% power to detect significant differences among the groups, satisfying the statistical requirements. Statistical analyses were performed using SPSS 25.0 (Chicago, SPSS). Categorical variables were described as frequencies (percentages) and assessed using the chi-square test. Continuous variables were presented as mean ± SD; t-tests and one-way ANOVA evaluated group differences, with *post hoc* tests for multiple comparisons. A *P*-value < 0.05 was considered statistically significant. Logistic regression analysis identified differences among influencing factors.

## Results

### Comparison of baseline data

The baseline comparison across the three groups revealed no significant differences in age (χ2 = 4.215, *P*=0.122), body mass index (BMI) (χ2 = 2.254, P=0.324), or infertility duration (χ2 = 1.256, *P*=0.534). However, the unexplained infertility rate was significantly higher in Group A than in Groups B and C (χ2 = 7.335, *P*=0.026). Regarding fertilization methods, there were no significant differences between the groups for *in vitro* fertilization (IVF) (χ2 = 3.553, *P*=0.171), intracytoplasmic sperm injection (ICSI) (χ2 = 5.707, *P*=0.095), or R-ICSI (χ2 = 0.616, *P*=0.736). For endometrial preparation methods, the natural cycle usage rate in Group C was significantly lower than in Groups A and B (χ2 = 6.733, *P*=0.035), while the ovulation induction cycle usage rate in Group C was significantly higher than in Groups A and B (χ2 = 10.511, *P*=0.001). No significant differences were observed in the use of hormone replacement therapy (χ2 = 1.923, *P*=0.382) or in endometrial thickness among the groups (χ^2^ = 0.754, *P*=0.684) (see **Table 1**).

**Table 1:** Comparison of basic data of high quality blastocysts from different sources of D3 blastomeres number during frozen-thawed embryo transfer cycle.

Index	≤6 cells Group A	7~9 cells Group B	≥10 cells Group C	*P*
No. of cycles (n)	66	381	127	
Age (y)	32.67 ± 3.9	31.94 ± 3.6	31.53 ± 3.7	0.122
Body mass index (kg/m^2^)	22.27 ± 3.2	22.08 ± 3.0	22.55 ± 3.0	0.324
Infertility duration (a)	3.96 ± 2.68	4.37 ± 3.0	4.19 ± 2.8	0.534
Types of infertility (%)				0.031
Primary infertility	40.9% (27/66)	32.5% (124/381)^a^	44.9% (57/127)	
Secondary infertility	59.1% (39/66)	67.5% (257381)^a^	55.1% (70/127)	
Infertility factor (%)				
Female factor	68.2% (45/66)	70% (282/381)	75.6% (96/127)	0.525
Male factor	9% (6/66)	6.0% (23/381)	11.8% (15/127)	0.095
Combined factors	7.6% (5/66)	11.4% (43/381)	4.7% (6/127)	0.078
Unexplained factors	7.6% (5/66)^A^	1.3% (5/381)	1.6% (2/127)	0.026
Multiple factors	7.6% (5/66)	7.3% (28/381)	6.3% (8/127)	0.912
Fertilization method (%)				
IVF	68.2% (45/66)	75.1% (286/381)	80.3% (102/127)	0.171
ICSI	31.8% (21/66)	22.3% (85/381)	18.1% (23/127)	0.095
RICSI	0	2.6% (10/381)	1.6% (2/127)	0.736
Inner membrane Scheme				
The natural cycle	27.3% (18/66)	24.4% (93/381)	14.2% (18/127)^a^	0.035
The Inducing ovulatory cycle	15.1% (10/66)	26% (99/381)	38.6% (49/127)^A^	0.001
Hormone replacement therapy	57.6% (38/66)	49.6% (189/381)	47.2% (60/127)	0.382
Endometrial thickness in ET (mm)	9.49 ± 1.74	9.62 ± 1.8	9.53 ± 1.6	0.684

P<0.01 indicates a highly significant difference and is represented by uppercase letters; P<0.05 indicates a significant difference and is represented by lowercase letters.

### Comparison of pregnancy outcomes

The comparison of pregnancy outcomes among the three groups revealed that the proportion of stage 5 or higher blastocysts in Group C (≥10 cells) was significantly greater than in Groups A and B (χ2 = 9.099, *P*=0.011). No significant differences were observed in the proportions of stage 3 and stage 4 blastocysts among the groups (χ2 = 4.413, *P*=0.110; χ2 = 0.554, *P*=0.758). Group C also had a significantly higher proportion of D5 blastocysts compared to Groups A and B (χ2 = 59.615, *P*=0.000), while Group A had a significantly higher proportion of D6 blastocysts than Groups B and C (χ2 = 59.615, *P*=0.000). Clinical pregnancy rates did not differ significantly among the groups (χ2 = 1.344, *P*=0.511). Similarly, no significant differences were found in multiple pregnancy rates (χ2 = 0.196, P=0.906) or implantation rates (χ2 = 1.344, *P*=0.511). The miscarriage rate in Group B was significantly higher than in Groups A and C (χ2 = 7.464, *P*=0.024). Finally, live birth rates were not significantly different across the groups (χ2 = 3.299, *P*=0.194) (see **Table 2**).

**Table 2:** Comparison of pregnancy outcomes in high-quality blastocysts from different sources of D3 blastomeres number during frozen-thawed embryo transfer cycles.

Index	≤6 cells Group A	7~9 cells Group B	≥10 cells Group C	*P*-value
No. of cycles(n)	66	381	127	
Blastocyst Stage				
Stage 3	22.7%(15/66)	18.6%(71/381)	11.8%(15/127)	0.110
Stage 4	68.2%(45/66)	71.4%(272/381)	68.5%(87/127)	0.758
Stage 5 and above	9.1%(6/66)	10%(38/381)	19.7%(25/127)^a^	0.011
Embryonic Age				0.000
D5	68.2%(45/66)	93.4%(356/381)^a^	99.2%(126/127)^A^	
D6	31.8%(21/66)	6.6%(25/381)	0.8%(1/127)	
Clinical pregnancy rate(%)	53%(35/66)	57.2%(218/381)	61.4%(78/127)	0.511
Multiple pregnancy rate(%)	0	5%(11/218)	3.8%(3/78)	0.906
implantation rate(%)	53%(35/66)	57.2%(218/381)	61.4%(78/127)	0.511
Miscarriage rate(%)	5.7%(2/35)	17.9%(39/218)^a^	9.4% (12/127)	0.024
Live birth rate(%)	94.3%(33/35)	82.1%(179/218)	83.3%(65/78)	0.194

P<0.01 indicates a highly significant difference and is represented by uppercase letters; P<0.05 indicates a significant difference and is represented by lowercase letters.

### Multivariate regression analysis

#### Multivariate logistic regression analysis of group A

A multivariate logistic regression model was developed with Group C as the reference category. This model incorporated variables such as blastocyst stage, embryo age, endometrial preparation protocol, infertility type, and miscarriage rate. The analysis revealed that blastocyst grading significantly influenced the outcome (RR = 0.063, 95% CI: 0.016–0.246, *P* < 0.001). Likewise, embryo age had a substantial effect on the outcome (RR = 4.309E+31, 95% CI: 3.366E+30–5.516E+32, *P* < 0.001). Conversely, the endometrial preparation protocol did not significantly impact the outcome (RR = 0.916, 95% CI: 0.265–3.157, *P* = 0.889), nor did infertility type (RR = 0.727, 95% CI: 0.165–3.213, *P* = 0.674). In contrast, miscarriage rate had a significant effect on the outcome (RR = 5.938, 95% CI: 2.189–16.105, *P* < 0.001) (see **Table 3**).

**Table 3:** The Results of multivariate Logistic Regression Screening of Group A.

Group	Variables	Regression Coefficient	Standard Error	*Wald chi-square value*	*P*	*RR*	95%*CI*
A	Intercept	-54.654	1.249	1913.603	0.000		
	Blastocyst Stage	-2.764	0.694	15.871	0.000	0.063	0.016-0.246
	Embryonic Age	72.841	1.301	3135.854	0.000	4.309E+31	(3.366E+30)-(5.516E+32)
	Endometrial preparation protocol	-0.088	0.632	0.020	0.889	0.916	0.265-3.157
	Types of infertility	-0.319	0.758	0.177	0.674	0.727	0.165-3.213
	Miscarriage rate	1.781	0.509	12.245	0.000	5.938	2.189-16.105

#### Multivariate logistic regression analysis for group B

A multivariate logistic regression model was constructed, with Group C serving as the reference, incorporating factors such as blastocyst stage, embryonic age, endometrial preparation protocol, infertility type, and miscarriage rate. The results for Group B showed that the blastocyst stage significantly impacted the outcome (RR = 0.162, 95% CI: 0.079–0.330, *P* < 0.001). Embryonic age also significantly influenced the outcome (RR = 21.935, 95% CI: 2.485–193.951, *P* = 0.005). Endometrial preparation protocol was significantly associated with the outcome (RR = 0.281, 95% CI: 0.149–0.529, *P* < 0.001). Furthermore, infertility type had a significant impact on the outcome (RR = 8.250, 95% CI: 3.783–17.991, *P* < 0.001), and miscarriage rate was likewise significantly associated with the outcome (RR = 2.864, 95% CI: 1.477–5.553, *P* = 0.002) (see **Table 4**).

**Table 4:** The Results of multivariate Logistic Regression Screening of Group B.

Group	Variables	Regression Coefficient	Standard Error	*Wald chi-square value*	*P*	*RR*	95%*CI*
B	Intercept	-1.543	1.235	1.560	0.212		
	Blastocyst Stage	-1.821	0.364	25.020	0.000	0.162	0.079-0.330
	Embryonic Age	3.088	1.111	7.725	0.005	21.935	2.485-193.591
	Endometrial preparation protocol	-1.270	0.323	15.424	0.000	0.281	0.149-0.529
	Types of infertility	2.110	0.398	28.143	0.000	8.250	3.783-17.991
	Miscarriage rate	1.052	0.338	9.700	0.002	2.864	1.477-5.553

## Discussion

In assisted reproductive technology (ART), selecting the optimal embryo for transfer is critical to achieving favorable pregnancy outcomes. Identifying embryos with the highest implantation potential continues to pose a significant challenge in managing patients undergoing *in vitro* fertilization and embryo transfer (IVF-ET). The findings of this study demonstrate that the number of cells in D3 embryos significantly influences the developmental stage and embryonic age of blastocysts. D3 embryos with the higher number of cells tend to develop into high-quality blastocysts earlier, specifically on day 5, and achieve a better blastocyst grade. By optimizing the selection of embryos based on embryonic age and different stages of blastocyst development, along with endometrial preparation protocols, it is possible to improve or achieve equally high clinical pregnancy rates and live birth rates.

Existing evidence suggests that the morphological grading of D3 embryos is correlated with the pregnancy outcomes of cleavage-stage embryos, as higher-quality embryos are more likely to result in successful implantation and pregnancy. However, the impact of D3 Cell Count on clinical pregnancy outcomes in frozen-thawed embryo transfer cycles involving high-quality blastocysts remains underexplored. Wang et al. (15) identified the number and growth of viable blastomeres as key indicators for predicting embryo developmental potential and clinical outcomes. High-quality embryos show that minimal damage during thawing does not impair their developmental potential or implantation rates. Sayme et al. (16) evaluated 195 oocytes from 40 patients undergoing antagonist Intracytoplasmic Sperm Injection (ICSI) treatment cycles. The cleavage-stage embryo arrangement was found to influence blastocyst formation rates. Specifically, embryos arranged in a tetrahedral configuration developed into a greater number of high-quality blastocysts compared to those with non-tetrahedral arrangements. The shorter time between ICSI and blastocyst formation in high-quality blastocysts suggests a strong correlation between embryo structural arrangement and the timing and quality of blastocyst formation. Liu et al. (17) noted that although C-grade embryos (>10 cells, symmetric blastomeres, and/or <20% fragmentation) possess relatively good developmental potential, A-grade embryos (6-10 cells, symmetric blastomeres, and/or <20% fragmentation) yield better clinical pregnancy outcomes. Xia et al. (18) recommended prioritizing high-quality blastocysts for selective frozen-thawed single embryo transfer. Despite the limited effect of blastocyst cleavage stage grading on pregnancy outcomes, it is preferable to choose embryos available on Day 2. If Day 3 embryos are not available, embryos with more than six cells on Day 2 should be prioritized for transfer. Similarly, Mi et al. (19) suggested that the number of cells on Day 2 influences blastocyst formation and clinical pregnancy rates. Consistent with our findings, the number of cells in D3 embryos significantly impacts the staging and embryonic age of blastocysts.

Wu et al. (20) demonstrated that in women under 35, live birth rates (LBR) significantly varied according to the number of cells on Day 3. Their findings indicate that as the number of Day 3 cells increases, LBR also rises following blastocyst transfer in younger women. Zhang et al. (21) identified a significant correlation between Day 3 morphological quality and the clinical pregnancy rate (CPR) and LBR in low-quality blastocysts. They suggested that when only low-quality euploid blastocysts are available for transfer, priority should be given to those derived from high-quality Day 3 embryos. Coticchio et al. (22) presented different results, showing that the formation rate of grade A blastocysts, based on inner cell mass (ICM) and trophectoderm (TE) morphology, was negatively correlated with the time to reach the blastocyst stage—indicating that higher-quality blastocysts formed in a shorter amount of time. Consistent with previous research, our study found that the number of cells in D3 embryos significantly influences blastocyst staging and embryonic age, with embryos containing more blastomeres often reaching high-quality blastocyst status earlier (on Day 5) and at a more advanced stage. High-quality blastocysts are associated with higher pregnancy and live birth rates. However, unlike previous studies, which included low-quality blastocyst transfers and observed significant differences in pregnancy outcomes between groups, our study focused exclusively on high-quality blastocysts, revealing no significant differences in pregnancy outcomes across groups.

Hu et al. (23) found no significant differences in pregnancy and birth outcomes between high-quality expanded blastocysts formed on Day 6 and those formed on Day 5. However, for low-quality blastocysts, the clinical pregnancy rate was higher on Day 5 compared to Day 6. In patients with inner cell mass (ICM) graded B or higher, the live birth rate was higher for blastocysts on Day 5 compared to those on Day 6. However, for patients with ICM graded C, there was no significant difference in live birth rates between blastocysts developed on Day 5 and Day 6. Ozgur et al. (24)found that fully expanded blastocysts (grade ≥3) with AA and BA scores had similar implantation rates, while those with AB scores had relatively lower rates. Logistic regression analysis indicated that female age, blastocyst age, blastocyst expansion score, trophectoderm score, and the number of frozen blastocysts were important predictors of clinical implantation. These findings are consistent with ours, as our study also found no significant differences in pregnancy outcomes between frozen-thawed cycles involving high-quality blastocysts formed on Day 5 and Day 6. In contrast, Coticchio et al. (22) reported that as blastocyst development time increased, implantation rates, sustained pregnancy rates, and live birth rates progressively decreased, even when adjusted for maternal age. Compared to Day 5 blastocysts, Day 6 blastocysts had significantly lower probabilities of implantation, clinical pregnancy, sustained pregnancy, and live birth (25). The divergence between these results and ours may be due to the fact that, although vitrification technology has advanced, it still cannot entirely prevent cell damage during freezing. We hypothesize that high-quality blastocysts (with more inner cell mass and trophectoderm cells) may better compensate for freezing-induced damage following thawed embryo transfer, but this assumption has not been directly proven by specific data or research in our study. Therefore, pregnancy outcomes between Day 5 and Day 6 high-quality blastocysts in frozen-thawed cycles show minimal differences. Additionally, embryo cell division requires time, and embryos with more blastomeres on Day 3 can more quickly increase the number of ICM and trophectoderm cells, allowing them to develop into high-quality blastocysts in a shorter time frame. This hypothesis aligns with our findings, as our study demonstrated that the number of cells in D3 embryos significantly impacts blastocyst staging and embryonic age.

The number of cells in D3 embryos is clearly associated with the formation of high-quality blastocysts. However, it remains uncertain whether this relationship is also influenced by factors such as fertilization methods, endometrial preparation protocols, or types of infertility. Martins et al. (26) proposed that differentially expressed proteins in semen might serve as biomarkers for diagnosing primary and secondary infertility. Their research further indicated that sperm maturation failure and immune responses are significant causes of both primary and secondary male infertility, affecting the development of high-quality embryos. In contrast, Abebe MS et al. (27) reported that the proportions of primary and secondary infertility in Africa are approximately equal, suggesting that embryo quality should be similar; however, this is not always observed. Similar to our study, this research found that the type of infertility is somewhat associated with the number of cells in D3 embryos, with patients suffering from secondary infertility showing higher pregnancy success rates. We believe this may be due to the fact that patients with secondary infertility have already experienced pregnancy and childbirth, demonstrating the potential for successful fertilization, embryo development, and pregnancy. However, patients with primary infertility may have other factors hindering pregnancy, which still require further investigation and elimination.

Jiang et al. (28) observed that fertilization rates, high-quality embryo rates, embryo implantation rates, clinical pregnancy rates, and live birth rates were comparable between early rescue ICSI and half-ICSI. Similarly, Ten et al. (29) noted that embryos obtained through conventional IVF had superior quality, as indicated by a higher number of grade A embryos, compared to those obtained via ICSI. Contrary to these findings, our study showed that the fertilization method did not affect the formation of high-quality blastocysts. Additionally, Madani et al. (30) reported no significant differences in embryo implantation rates, ongoing pregnancy rates, miscarriage rates, or live birth rates among natural cycles, ovulation induction cycles, and hormone replacement cycles, which contrasts with our results. Our study found that inappropriate endometrial preparation protocols may lead to poorer pregnancy outcomes, and optimizing endometrial protocols may improve pregnancy outcomes.

This study underscores the critical role of selecting suitable embryos for transfer in assisted reproductive technology, as it has a significant impact on pregnancy outcomes. Multivariate regression analysis identified several key factors influencing the number and quality of embryo cells. There is a significant correlation between blastocyst stage, embryonic age, and the number of cells in D3 embryos, particularly in Day 5 embryos and more advanced-stage blastocysts. The increase in miscarriage rates may be related to the number of cells in D3 embryos, and insufficient endometrial preparation partially affects clinical pregnancy outcomes. The type of infertility is also somewhat associated with the number of cells in D3 embryos, with patients suffering from secondary infertility showing higher pregnancy success rates.

A key limitation of this study is that the sample was obtained from a single reproductive center. Consequently, despite the large sample size and the rigorous study design, the results may not fully generalize to other regions or institutions. Variations in patient demographics, treatment protocols, and laboratory conditions across different reproductive centers could influence embryo transfer success rates and pregnancy outcomes. As a result, the external validity of the findings may be limited. To strengthen the generalizability of these results, future studies should be conducted across multiple centers to verify the findings in diverse clinical settings.

In summary, the results of this study demonstrate that D3 cell count and related factors play a critical role in pregnancy outcomes during frozen-thawed high-quality blastocyst transfer cycles. Optimizing embryo development time, selecting blastocysts at different stages, and refining endometrial preparation protocols may help improve clinical pregnancy and live birth rates.

## Data Availability

The original contributions presented in the study are included in the article/supplementary material. Further inquiries can be directed to the corresponding author.
